# Prevalence of syphilis, neurosyphilis and associated factors in a cross-sectional analysis of HIV infected patients attending Bugando Medical Centre, Mwanza, Tanzania

**DOI:** 10.1186/s12889-020-09984-9

**Published:** 2020-12-04

**Authors:** Adeodatus Haule, Betrand Msemwa, Evarista Mgaya, Peter Masikini, Samuel Kalluvya

**Affiliations:** 1 Department of Medicine, Catholic University of Health and Allied Sciences Bugando, Mwanza, Tanzania; 2 Laboratory, Catholic University of Health and Allied Sciences Bugando, Mwanza, Tanzania; 3 Department of Ophthalmology, Bugando Medical Centre, Mwanza, Tanzania

**Keywords:** Human immunodeficiency virus, Syphilis, Neurosyphilis, Human immunodeficiency virus- *Treponema pallidum* co-infection

## Abstract

**Background:**

HIV-syphilis co-infection can enhance the rapid progression of early or late latent syphilis to neurosyphilis and can cause catastrophic neurological complications. In studies in Mwanza, syphilis affects ~ 8% of healthy outpatients and studies done in the 1990s have suggested that up to 23.5% of HIV-syphilis co-infected patients also have neurosyphilis.

**Methodology:**

This was a cross sectional study in which adult HIV infected patients who were hospitalized or attending the outpatient Care and Treatment Clinic (CTC) were interviewed using a structured questionnaire and screened for syphilis using serum Treponema Pallidum Hemagglutination Assay (TPHA). Blood was also taken for CD4+ T cells and viral load. Those who were found to have syphilis underwent neurological examination for any neurologic deficit and were offered a lumbar puncture.

**Results:**

The prevalence of syphilis in HIV infected patients was found to be 9.6%. The majority of patients were female (72.5%) and median age was 42 years [interquartile range, 32–50]. Most patients were on ART (99.4%). In the study population of 1748 participants, 9.6% were TPHA positive; the majority (89.2%) reported not knowing their syphilis status and not previously been treated. One hundred and forty-one participants with syphilis had neurological examinations performed. Four of these had abnormal findings that necessitated a lumbar puncture. Neurosyphilis was confirmed in one patient (0.7%).

**Conclusion:**

The high prevalence of syphilis in HIV infected patients indicates that there is a need to increase efforts in targeting this population to reduce sexually transmitted infections. Screening for syphilis should be done for all HIV patients given the high prevalence of the infection and the risk that aggressive forms of neurosyphilis can occur despite recovery of CD4+ T cell counts in untreated syphilis.

## Background

In spite of effective preventive and treatment options, syphilis is still a global health problem causing high morbidity. It is estimated that about 10 million people worldwide are infected each year with a global prevalence of about 0.5% [1]. In sub-Saharan Africa, the burden has dropped from 6% to around 1% in the past 50 years [1, 2]. In Tanzania the prevalence of syphilis ranges from 2.5 to 8% in antenatal clinics and community surveys, respectively [3, 4]. In sub-Saharan Africa heterosexuals are most highly affected [5].

Human Immunodeficiency Virus (HIV)-*Treponema pallidum* co-infection represents an important problem with serious clinical implications. The genital ulcers caused by primary syphilis infection facilitate acquisition of HIV. The genital ulcers are usually accompanied by inflammatory cells that express HIV co-receptors, facilitating establishment of initial HIV infection in the genital mucosa [5]. Additionally the presence of syphilis co-infection in HIV infected patients increases the HIV viral load [6]. HIV-*Treponema pallidum* co-infected individuals tend to develop neurosyphilis more frequently as compared to people with *T.pallidum* infection alone [5]. HIV infection has also been associated with syphilis treatment failure [7].

The prevalence of neurosyphilis in untreated early syphilis among HIV positive patients has been reported to be as high as 23.5% in Spain [8]. Likewise 24.6% of HIV positive patients were reported to have neurosyphilis in a study done in Canada and development of neurosyphilis was significantly associated with CD4 less than 500 cells/μl and uncontrolled viremia [9]. Little is known about the magnitude of the problem of HIV-syphilis in Africa [10]. Hence the study was designed to determine the prevalence of syphilis, neurosyphilis and associated factors among HIV positive patients at Bugando Medical Centre in Tanzania.

## Methods

### Sample population, data collection, and laboratory testing and data analysis

The study was conducted at Bugando Medical Centre’s medical department. Bugando Medical Centre is one of four zonal hospitals in Tanzania; it serves a catchment population of about 15 million people with a bed capacity of about 900. The Bugando HIV outpatient clinic was started in 2004 and at present has more than 15,000 HIV-infected patients enrolled with over 5000 on antiretroviral therapy. The centre cares for all patients diagnosed from within the hospital and those sent from catchment facilities. All patients who are newly diagnosed with HIV are routinely screened for opportunistic infections and Hepatitis B and C. In the first 3 months of 2017 newly diagnosed HIV patients were screened for syphilis and 36/436(8.3%) were Venereal Disease Research Laboratory (VDRL) positive. At the time of study screening for neurosyphilis was not done routinely.

This was an observational cross-sectional hospital based study. The study involved all HIV positive adult patients who were admitted to the Bugando Medical Centre medical wards and those who were seen at the CTC. All HIV positive patients who were 18 years and above and had tested positive for *T. pallidum* in the 3 months prior to study initiation or during the study period were included. All HIV negative patients were excluded.

The convenience sampling method was used; all HIV positive patients admitted to the hospital or being seen at the CTC over the 4 month period were approached to offer them the opportunity to participate in the study. All participants provided written consent.

A minimum sample size of 138 patients was calculated by means of the Kish Leslie formula using a predicted prevalence of neurosyphilis among HIV positive patients of 10% [8, 9].

Participants were interviewed using a structured questionnaire which included age, sex, marital status, education, and a mental status examination. Five millilitres of blood were drawn by trained personnel into Ethylene Diamine Tetra Acetic Acid (EDTA) bottles and tested for *Treponema pallidum* antibodies by Treponema Pallidum Hemagglutination Assay (TPHA) as well as for CD4 count and viral load. Participants with positive results underwent a thorough neurological examination including cognitive assessment, fundoscopic examination and subjective audiometry (Weber and Rinne tests). These patients were also examined for sensory, motor, gait and balance deficits. Participants with neurologic abnormalities were offered a lumbar puncture by trained personnel. Neurosyphilis was defined according to the Centers For Disease Control and Prevention (CDC) criteria as serum treponemal test TPHA positive plus neurological features as well as CSF-VDRL positive and /or 20 WBC/microlitre or more in CSF without other clear cause [11]. Patients discovered to have syphilis or neurosyphilis were referred to their attending physician who provided treatment as per CDC guidelines. The first line treatment for neurosyphilis was intravenous (IV) benzyl penicillin 18 to 24 MU divided in 3 to 4 MU every 4 h for 10 to 14 days; second line treatment was procaine penicillin G 2.4 MU daily with probenecid 500 mg four-hourly for 10 to 14 days. Patients were followed up for 30 days beginning from the day of treatment to assess clinical outcome after treatment [11, 12].

Data were collected using a coded questionnaire and entered into Microsoft Excel. Data was analysed using STATA version 13 (College Station, Texas). All continuous variables were summarized as medians with interquartile ranges, while categorical variables were summarized as proportions or percentages using chi-squared tests. Univariable followed by multivariable logistic regression analyses were used to determine factors associated with syphilis. Any factor with a *p*-value of < 0.3 on univariable logistic regression analysis was included in the multivariable model. A p-value of less than 0.05 was considered to be statistically significant in the final model.

Ethical clearance was obtained from the Catholic University of Heath and Allied Science / Bugando Medical Centre Joint Ethics and Review Committee with certificate number CREC/242/2017. Written informed consent was obtained from each patient or the patient’s next of kin for those unable to consent for themselves because of illness or severe cognitive impairment. Results were communicated to the treating physician immediately and a copy of the results was placed in the patient’s file.

## Results

In this study, a total of 1748 participants were screened for syphilis and 167 (9.6%) were found to be serum TPHA positive. More than half, 1008 (57%) of the studied participants had enrolment CD4 counts of > 350 cells/uL and 1333 (76.3%) had viral loads of less than 50 copies /ml. Details of the 167 study participants who were TPHA positive are found in Table 1. Females comprised a large number with positive serum TPHA (72.5%) and the majority of those with TPHA positivity were aged 40–64 years (100/167, 59.9%). The *T.pallidum* seropositive group was significantly older than the TPHA negative group as shown in Table 2. The TPHA positive group also had more monogamously married people and more people with only primary education. Vendors constituted the largest occupational group comprising 104 (62.3%) among those who were *T.pallidum* seropositive.

**Table 1:** General characteristics of the 1748 HIV positive study participants

***Variable***	Frequency	Percent or Median (IQR)
***Gender***		
**Male**	496	28.4
**Female**	1252	71.6
***Age***	1748	42 [34–50]
**18–39**	729	41.7
**40–64**	966	55.3
**65–85**	53	3.0
***Marital status***		
**Single**	543	31.1
**Married(monogamy)**	940	53.8
**Polygamy**	258	14.8
**Window**	7	0.4
***Occupation***		
**Vendor**	1114	63.7
**Peasant**	338	19.3
**Fisherman**	10	0.6
**Employed**	211	12.1
**House wife**	75	4.3
***Residence***		
**Mwanza city**	1552	88.8
**Mwanza city**	196	11.2
**Outside**		
***Education***		
**Illiterate**	126	7.2
**primary**	1170	66.9
**Secondary**	373	21.3
**College**	60	3.4
**University**	19	1.1
***Reports being told to have syphilis***		
**No**	1695	97.0
**Yes**	53	3.0
***Reports previously being treated for syphilis***		
**No**	17	32.1
**Yes**	36	67.9
***Reports penicillin injection***		
**One**	4	11.1
**Three**	32	88.9
***History of gental lesion p***		
**No**	1698	97.1
**Yes**	50	2.9
***Number of lesion***		
**One**	15	30
**> two**	35	70
***Duration of lesion***		
**< year**	29	58.0
**2-3 year**	11	22.0
**4-6 year**	4	8.0
**> 6 years**	6	12.0
***Rashes on palm and sole***		
**No**	1740	99.5
**Yes**	8	0.5
***Treatment for rashes***		
**No**	3	37.5
**Yes**	5	62.5
***ART use***		
**Not on ART**	1	0.1
**ART use**	1747	99.9
***Serum TPHA***		
**Negative**	1581	90.5
**Positive**	167	9.6
***CD4 Cell/μl***	1748	526 [362–706]
**< 350**	306	17.5
**> 350**	1008	57.7
**missing**	434	24.8
***Viral load (copies/uL)***	1748	19 [19–20]
**< 50**	1333	76.3
**50–999**	78	4.5
**> 1000**	84	4.8
**Missing**	253	14.5

**Table 2:** Socio-demographic and reported clinical characteristics of 167 HIV-positive individuals who were serum *T.pallidum* seropositive compared to 1581 *T.pallidum* seronegative Participants

Variable	TPHA Positive		X^**2**^	***P***-Value
	YES N (%)	NO N (%)		
**Gender**				
**Male**	46 (27.5)	450(28.5)	0.063	0.802
**Female**	121(72.5)	1131(71.5)		
**Age**				
**18–39**	58(34.7)	671(42.4)	6.349	0.044
**40–64**	100(59.9)	866(54.8)		
**65–84**	9(5.4)	44(2.8)		
**Marital status**				
**Single**	38(22.8)	505(31.9)	15.73	< 0.001
**Married(monogamy)**	94(56.3)	846(53.3)		
**Widow**	32(19.2)	226(14.3)		
**Polygamy**	3(1.8)	4(0.25)		
**Education**				
**Illiterate**	29(17.4)	97(6.1)	34.53	< 0.001
**Primary**	112(67.1)	1058(66.9)		
**Secondary**	21(12.5)	352(22.3)		
**College**	3(1.8)	57(3.6)		
**University**	2(1.2)	17(1.1)		
**Residence**				
**Mwanza city**	148(88.6)	1404(89)	0.005	0.944
**Outside Mwanza city**	19(11.4)	177(11)		
**Occupation**				
**Vendor**	104(62.3)	1010(63.9)	19.28	< 0.001
**Peasant**	44(26.4)	294(18.6)		
**Fisherman**	2(1.2)	8(0.5)		
**Employed**	6(4.0)	205(13.0)		
**House wife**	11(6.6)	64(4.1)		
**Reports previous history of syphilis**				
**No**	149(89.2)	1546(97.8)	37.68	< 0.001
**Yes**	18(10.8)	35(2.2)		
**Reported previous treated syphilis**				
**No**	6(33.3)	11(0.7)	0.02	0.888
**Yes**	12(66.7)	24(1.5)		
**Reports penicillin injection**				
**One**	2(16.6)	2(8.30)	0.563	0.453
**Three**	10(83.3)	22(91.7)		
**History of genital lesion**				
**No**	153(91.6)	1545(97.7)	20.27	< 0.001
**Yes**	14(8.4)	36(2.3)		
**Number of lesion**				
**One**	10(71.4)	5(13.9)	15.89	< 0.001
**Two or more**	4(28.6)	31(89.1)		
**Duration of lesion**				
**Less than a year**	9(64.3)	20(55.6)	0.536	0.911
**Two to three year**	3(21.5)	8(22.2)		
**4 to six year**	1(7.1)	3(8.3)		
**More than six year**	1(7.1)	5(13.9)		
**Rashes on palms and soles**				
**No**	164(98.2)	1576(99.6)	7.264	0.007
**Yes**	3(1.8)	5(0.3)		
**Treatment for rashes**				
**No**	3(100.0)	0(0)	8	0.005
**Yes**	0(0.0)	5(100)		
**ART use**				
**No**	1(0.6)	0(0)	9.473	0.002
**Yes**	166(99.4)	1681(100)		
**Duration of ART use**				
**Less 6 month**	3(1.8)	0	1400	< 0.001
**6 month to 2 years**	25(15.0)	0		
**Above 2 yrs**	111(66.5)	0		
**Missing**	28(16.8)	1681		
**Viral load(copies/ml)**				
**Less than 50**	130(77.8)	1203(79.1)	1.424	0.7
**50–999**	7(4.2)	71(4.5)		
**1000 or more**	10(6.0)	74(4.7)		
**Missed viral load**	20(12.0)	233(14.7)		
**CD4 cell/uL**				
**Less than 350**	35(21.0)	271(17.1)	1.638	0.441
**350 and above**	94(56.3)	914(57.8)		
**Missing**	38(22.7)	396(25.15)		
**CSF WBC/UL**				
**Less than or equal 5**	4(100.0)			
**Greater 5**	0(0.0)			
**CSF Treponema antibody**				
**Negative**	3(75.0)			
**Positive**	1(25.0)			

In 167 participants, 18 (10.8%) reported a previous history of syphilis and among them 12 (66.7%) reported prior history of treatment with penicillin injections are found in Table 2. Of these, 10 (83.3%) had 3 penicillin injection doses and 2 (16.7%) received a single dose. Only 14 participants (8.4%) reported a prior history of chancre while the majority (10, 71.4%) reported a single genital lesion. In nine participants (64.3%) the chancre had occurred in the past year. Only 3 (1.8%) participants had reported painless rashes on the palms or soles and none of these received treatment. All except for one participant were on ART 166 (99.4%).

Details of the 141 patients who underwent neurological examination are shown in Table 3. Most of the participants (138, 97.9%) had no cognitive impairment while one had mild and two had severe impairments. Only two participants (1.5%) had blurred vision and, after further examination, one was found to have a cataract in the left eye and the other had a corneal ulcer. Both of these patients were managed by the ophthalmologist accordingly. No features of syphilis of the eye were noted. Most of patients were on ART and the majority had been on ART for more than 2 years (111, 66.5%).s. A large number (130, 77.8%) of those who were seropositive for *T.pallidum* had a suppressed viral load of less than 50 copies/ml and only a small number of participants (10, 6%) had a viral load of more than 1000 copies/uL. More than half of the participants had CD4+ T cells above 350 cells/uL (94, 56.3%).

**Table 3:** Clinical findings among 141 HIV positive patients who were seropositive for *T.pallidum* and returned for further evaluation Mwanza Tanzania

Variable	Frequency	Percent
**MMS**		
24–30	138	97.9
18–23	1	0.7
0–17	2	1.4
**Neck pain noted on examination**		
Yes	0	0
No	141	100
**Noted reduced vision**		
Yes	2	1.5
No	139	98.5
**Seen with confusion**		
Yes	2	1.5
No	139	98.5
**Had noted with fever**		
Yes	1	99.3
No	142	0.7
**Gait**		
Normal	139	98.5
Abnormal	0	–
Bedbound	2	1.5
**Sight loss right**		
Absent	141	100.0
Present	0	–
**sight loss left**		
Absent	141	100.0
Present	0	–
**Uveitis**		
Absent	141	100.0
Present	0	–
**Argyll-Robertson pupil**		
Absent	141	100.0
Present	0	–
**Hearing loss right**		
Absent	140	99.3
Present	1	0.7
**Hearing loss left**		
Absent	140	99.3
Present	1	0.7
**Type of hearing loss**		
No deficit	140	99.3
Sensorineural	1	0.7
Conductive	0	–
**Romberg’s sign**		
Negative	141	100.0
Positive	0	–
**Position sense**		
Normal	141	100.0
Abnormal	0	–
**Vibration sense**		
Normal	141	100.0
Abnormal	0	–
**Reflexes**		
Normal	141	100.0
Reduced	0	–
Exaggerated	0	–

Two patients were noted to have confusion (1.5%) of whom one had fever as well (0.7%). Both of these were bedbound and their gait could not be assessed. No participant was noted to have sight loss, uveitis, or Argyll-Robertson pupils. Two of the participants were noted to have hearing loss. One had right-sided sensorineural hearing loss while all other neurological features were normal. The other had reduced cognition, thus, it was difficult to assess the type of hearing loss. The bed bound patients did not have Romberg or vibration sense tests done. All the other patients had negative Romberg’s tests and a normal vibration sense. All had normal reflexes.

There were four participants who were seropositive for *T. pallidum* with neurological abnormalities who underwent lumbar puncture after counselling and consent as shown in Table 5. One patient was CSF–VDRL positive and had CSF-WBC < 5 cells/uL. Three patients had negative CSF–VDRL and CSF-WBC < 5 cells/uL. In the univariable logistic regression analysis, several factors were statistically significantly associated with seropositivity for *T.pallidum* including older age (Odds ratio (OR) 2.37 [95% confidence interval (CI), 1.10–5.09], *p* = 0.027), being widowed (OR 1.88 [1.50–3.90], *p* = 0.013), polygamy (OR 9.97, [2.15–46.16], *p* = 0.003), prior history of genital chancre (OR 3.93, [2.07–7.44], *p* <  0.001), and previous history of syphilis (OR 5.34 [95% CI, 2.95–9.65], *P* <  0.001) details of the finding is found in Table 4. On multivariable logistic regression analysis, only polygamy (OR 8.51 [1.71–42.37], *p* = 0.009) and previous history of syphilis (OR 3.5[1.75–7.01], *p* <  0.001) remained independently associated with syphilis co-infection.

**Table 4:** Univariable and multivariable analysis for factors associated with syphilis co- infection

***Variable***	***Univariable***		***Multivariable***	
	OR (95%CI)	***P*** Value	OR(95% CI)	***P*** Value
***Sex***				
**Male**	1			
**Female**	1.04 (0.73–1.50)	0.802		
***Age***				
**18–39**	1		1	–
**40–64**	1.34(0.95–1.87)	0.094	1.13(0.78–1.63)	0.514
**65–85**	2.37(1.10–5.09)	0.027	1.34(0.57–3.13)	0.502
***Marital status***				
**Single**	1		1	–
**Married**	1.48(1.00–2.29)	0.052	1.42(0.94–2.14)	0.091
**Widow**	1.88(1.50–3.90)	0.012	1.47(0.86–2.52)	0.158
**Polygamy**	9.97(2.15–46.16)	0.003	8.51(1.71–42.37)	0.009
***Education***				
**Illiterate**	1		1	–
**Primary**	0.35(0.22–0.56)	< 0.001	0.38(0.23–0.61)	< 0.001
**Secondary**	0.2(0.19–0.36)	< 0.001	0.26(0.14–0.50)	< 0.001
**College**	0.18(0.05–0.60)	0.006	0.29(0.08–1.06)	0.062
**University**	0.39(0.86–1.80)	0.230	0.77(0.16–3.67)	0.739
***Occupation***				
**Vendor**	1		1	–
**Peasant**	1.45(1.00–2.17)	0.051	1.15(0.77–1.72)	0.489
**Fisherman**	2.43(0.51–11.58)	0.226	1.76(0.29–10.54)	0.535
**Employed**	0.28(0.12–0.66)	0.003	0.33(0.14–0.78	0.011
**House wife**	1.16(0.85–3.26)	0.134	1.57 (0.79–3.13)	0.202
***Chancre***				
**No**	1		1	–
**Yes**	3.93(2.07–7.44)	< 0.001	2.1(0.96–4.58)	0.063
***Rashes***				
**No**	1		1	–
**Yes**	5.76(1.37–24.34)	0.017	2.02(0.41–9.92)	0.388
***Told syphilis***				
**No**	1		1	–
**Yes**	5.34(2.95–9.65)	< 0.001	3.5(1.75–7.01)	< 0.001
***Previously treated syphilis***				
**No**	1			
**Yes**	0.92(0.27–3.08)	0.888		
***Viral load (copies/uL)***				
**< 50**	1			
**50–999**	0.91(0.41–2.02)	0.822		
**1000 or more**	1.25(0.63–2.48)	0.522		
**Not tested**	0.79(0.49–1.30)	0.358		

One patient among four had neurosyphilis confirmed with CSF-VDRL positive while CSF –WBC < 5. The other three patients did not meet the criteria of neurosyphilis in HIV patients according to CDC recommendations. Table 5 below shows the details of the CSF finding after the lumbar puncture in the four patients who underwent a lumbar puncture.

**Table 5:** Clinical Descriptions of Four seropositive for *T.pallidum* patients who had neurologic abnormalities and underwent lumbar puncture

***Patient number***	***1***	***2***	***3***	***4***
**Gender**	Male	Female	Female	Female
**Age in (years)**	50	65	57	30
**ART use**	Not on ART	yes	yes	yes
**Duration of ART**	–	4 months	2 years	7 year
**Neurologic features**	Headache Confusion fever	-Disturbed memory	Confusion Hearing loss left Disturbed memory and cognition	Hearing loss right ear which is sensorineural No ear discharge Normal other system
**CD4+/uL**	412	–	219	270
**Viral load (copies/Ul)**	Not tested	Not tested	19	19
**CSF finding**	-Positive VDRL -WBC < 5/uL Glucose low(2.99 mmol) -Normal protein -Cryptococcal antigen negative(CRAG) -Clear fluid with negative gram stain and culture -Opening pressure extremely high	Negative VDRL -WBC < 5/Ul -Normal glucose -Normal protein -Negative CRAG -Negative culture	Negative VDRL WBC < 5 cell/Ul Normal glucose Normal protein Negative culture Negative CRAG	-Negative VDRL WBC < 5cell/uL Normal glucose Normal protein Negative culture
**Diagnosis**	Neurosyphilis with meningoencephalitis	Presumed HIV Encephalopathy	Presumed HIV Encephalopathy	Hypoacusis due to other causes
**Outcome.**	Admitted to the ward and started on Ceftriaxone 2 g IV BD. Died after three days in the course of treatment in the ward.	Alive, cognition deficit improving on ART.	Discharged home alive but later died at home.	Alive and hearing deficit has improved

## Discussion

In this study of HIV positive participants, 9.6% were seropositive for *T.pallidum*. The finding was similar to a prevalence rate of 10.0% reported in a study done in Uganda [13] and another in Ethiopia where 9.8% out of the 306 HIV positive patients were found to be seropositive for *T.pallidum* [14]. The seroprevalence that we observed was lower than that found in Ghana (14.8% of 284 HIV-infected participants) and higher than a study done in Rwanda in which 4.8% of 482 HIV infected participants were syphilis seropositive.

People who reported being married to one person were significantly more frequently seropositive for *T.pallidum* (56.3% versus 53.5% in the TPHA negative). This finding is similar to study results from Uganda [13] and Ethiopia [14] and may be because of concurrency of partners. Specifically, Kenyon et al reported that male partner concurrency in which men had an average of five concurrent partners was significantly associated with a high prevalence of syphilis [15]. People who were TPHA positive were significantly more likely to have only a primary education perhaps suggesting that they may have a lower knowledge of preventive measures against sexually-transmitted infections. This finding was contrary to the study done in Ethiopia in which having a secondary education was associated with TPHA positivity. Employed persons were less likely to have seropositive *T.pallidum* in our study. Only 10% of the patients who were seropositive for *T.pallidum* reported a previous history of syphilis. Syphilis is not screened for routinely among people living with HIV and perhaps at times not thought to be an important problem. Similarly, high rates of undiagnosed syphilis have been reported in Ethiopia. For those who had syphilis, only two-thirds (66.7%) received treatment. The majority received three intramuscular penicillin doses according to CDC recommendation as also reported by Katz and colleagues [16].

Most patients who were seropositive for *T.pallidum* had CD4+ T cells above 350 cells/uL (56.3%) and viral load levels less than 50 copies/mL (77.8%) contrary to our hypothesis that those with *T.pallidum* seropositivity would have low CD4+ T cell counts. We did not find an association between syphilis and CD4 counts or viral loads. This might be because most of the patients in our study were on ART and in a latent stage of syphilis. Our results contrast with the study in the US by Kate et al which reported that syphilis reduces CD4+ T cells and increases viral load [6], particularly in those with secondary syphilis on ART and those with syphilis not on ART. We also found that the majority of participants who were seropositive for *T.pallidum* reported no prior history of genital lesion (97.1%), possibly due to the painless lesions of syphilis that might go unnoticed in primary stages. A study in Spain similarly found that few patients who were seropositive for *T.pallidum* reported a past genital lesion [8].

Among the 141 participants who were serum TPHA positive and returned to the clinic for neurological assessment, 3 participants had cognitive impairment. One of those with cognitive impairment also had hearing loss. The second patient had hearing loss alone with no other symptoms. The third patient had fever, headache, and altered mental status. The patients were also assessed for any sign of meningeal irritation but no one was positive. In addition, eye examinations failed to identify a patient with typical features of ophthalmic syphilis. Patients were also examined for gait, unilateral weakness, and sensory modalities; they were all found to be normal. This is in contrast with the study done in the US by Katz et al which found 12 patients with neurosyphilis, of whom 4 had eye problems, 3 had an altered mental status and 5 had unilateral weakness [16]. Of note, a major difference between the Tanzanian and the US study is that not all US patients were on ART, whereas all but one of the Tanzanian study patients were on ART.

Among the 141 screened by examination, only 4 (2.8%) had neurological symptoms necessitating a lumbar puncture to assess for neurosyphilis. This was in accordance with expert guidelines recommending lumbar punctures only for those with neurological abnormalities but not for all seropositive for *T.pallidum* plus HIV-positive patients [11, 12, 17]. In our study, among the 4 participants with syphilis who underwent a lumbar puncture, one was confirmed to have neurosyphilis. That person had no prior history of syphilis and was not on ART. The CD4+ T cell count was 412 cells/uL. The patient was treated with daily intravenous ceftriaxone as per the CDC guidelines but died after 3 days on the ward after a rapid neurologic deterioration. This patient might have suffered from the meningoencephalitic form of neurosyphilis which has a swift progression with a poor prognosis in HIV positive patients. This form of neurosyphilis was seen in one case study in a HIV-positive patient who presented with abnormal behaviour [18]. In HIV positive patients, even those with normal CD4+ T cell counts, immune cells may have altered function increasing the risk of syphilitic meningoencephalitis [19]. Likewise a study in the US found 16 neurosyphilis patients who were identified with only CSF-VDRL positivity among 50 patients with neurosyphilis [20]. By contrast, in the study done in Spain, all patients who had a diagnosis of neurosyphilis presented with mild headache, had no prior history of treatment, and improved after therapy [8]. Patients, who were negative for neurosyphilis, were all on ART and reported no history of prior treatment for syphilis. This may have occurred because ART use reduces the chances of having neurosyphilis to a level comparable to that of HIV-uninfected people with syphilis [21].

In our study, we found a prevalence of neurosyphilis of only 0.7% for all who were examined for neurologic features. This prevalence was surprisingly low given the findings of other studies that have suggested the prevalence could be as high as 25%. This low prevalence may be due either to ART use in most patients or possibly to having been previously treated with antibiotics, for a different indication, that have activity against *Treponema pallidum*. Our findings on neurosyphilis fit with a systemic review of neurosyphilis in Africa [22], which found only two patients with meningitis (3.3%). Another study done in Brazil had shown the prevalence of neurosyphilis to be 1% among HIV positive patients with neurologic features [23]. In contrast, a study by Alverez et al showed that 23.5% *T.pallidum* seropositive patients who were not on ART had neurosyphilis [8]. Therefore, our study documents an important and encouraging finding that rates of neurosyphilis are lower in our setting than previously reported. This may be due to the expanding use of ART and also to the use of antibiotics, some of which may be excessive, but may serve inadvertently to treat *T.pallidum* seropositive patients and prevent neurosyphilis.

Our study had some limitations. It was difficult to know if patients had been previously treated for syphilis with other agents that are active against syphilis like ceftriaxone or doxycycline because of the lack of electronic data keeping. In our study, we did not perform a lumbar puncture in patients without neurologic features and, hence, we may have missed asymptomatic neurosyphilis. We were not able to determine whether a patient may have had seroreversion of syphilis as the reagent used was only qualitative and usually stays positive for life. Risk factors for neurosyphilis could not be determined due to the very small number. HIV-positive patients with neurosyphilis may die rapidly and this cross-sectional study would not have found those patients.

## Conclusion

We found a high percentage of seropositivity for *T.pallidum* in HIV positive participants in which one out of ten people was affected yet a very low prevalence of neurosyphilis. This argues for the need for sexually transmitted infection (STI) screening especially syphilis with a specific focus on HIV positive patients. Factors associated with syphilis were having multiple partners and having a low level of education. We also observed a high rate of untreated syphilis among HIV-positive patients. Only 10.8% of HIV-positive patients who had syphilis were aware of their diagnosis which highlights the importance of prioritizing screening and treatment. We, therefore, recommend that screening for syphilis should be provided to all HIV positive individuals and that those with untreated syphilis should receive three doses of penicillin. For those with syphilis involving central nervous system manifestations, urgent treatment with benzyl penicillin or ceftriaxone according to the National Standard treatment guidelines or CDC recommendations is required. Screening and treating syphilis in all HIV positive patients should be routinely done as the disease can present as fulminant meningoencephalitis and, consequently, death. In HIV positive patients with neurological manifestations, neurosyphilis should be considered as one of the diagnoses. We recommend further studies being done among HIV positive patients with neurological manifestations who are admitted to the hospital to rule out neurosyphilis.

## Data Availability

The datasets generated and/or analysed during the current study are available and can be accessed for further use in the researchdata@springernature.com

## Abbreviations

ART: Antiretroviral therapy
BMC: Bugando Medical Centre
CDC: Center for Disease Control
CD4: Cluster differention
CREC: CUHAS /BMC Joint Ethics and Review Committee
CSF: Cerebrospinal fluid
CTC: Care and treatment clinics
CUHAS: Catholic University of Health and Allied Sciences
HIV: Human immunodeficiency Virus
TPHA: Treponema pallidum Haemoglutination Assay
VDRL: Venereal disease research laboratory
WBC: White blood cell
